# Screw dislocation in a Rashba spin-orbit coupled $\alpha$-$T_3$ Aharonov–Bohm quantum ring

**DOI:** 10.1038/s41598-024-61889-4

**Published:** 2024-05-16

**Authors:** Mijanur Islam, Saurabh Basu

**Affiliations:** Department of Physics, Indian Institute of Technology Guwahati, Guwahati, Assam 781039 India

**Keywords:** Condensed-matter physics, Quantum physics, Physics

## Abstract

In this paper we investigate the effect of a topological defect, such as a screw dislocation in an $\alpha$-$T_3$ Aharonov–Bohm quantum ring and scrutinized the effects of an external transverse magnetic field and Rashba spin-orbit coupling therein. The screw dislocation yields an effective flux which reshape the periodic oscillations in the persistent current in both charge and spin sectors, with a period equal to one flux quantum. Moreover, they suffer a phase shift proportional to the degree of dislocation, and include scattering effects due to the dislocation present in the system. Such tunable oscillation of the spin persistent current highlights applications of our system as potential spintronic devices. Further, the behaviour of the current induced by the Burgers vector ($b^z$) which denotes the strength of the dislocation is investigated in the absence and presence of an external magnetic field. In both the scenarios, an almost linear decrease in the current profile as a function of the Burgers vector is observed. Notably, without the external magnetic field, the Burgers current suffers a back flow for $\alpha =1$ (dice lattice), while in the presence of the external magnetic field, for other values of $\alpha$ (e.g., $\alpha =0.5$) this back flow occurs for a specific value of $b^z$. Additionally, the presence of the distortion induces a chirality effect, giving rise to an additional chiral current even in the absence of an external field. Furthermore, in the absence of field, the Burgers spin current initially rises, attains a maximum before diminishing as $b^z$ is enhance for all values of $\alpha$. However, such a non-monotonicity in the Burgers spin current is conspicuously non-existent in the presence of an external field. The chiral current discussed above may hold important applications to spintronics.

## Introduction

In recent years, quantum rings (QRs) have garnered significant attention due to their diverse technological applications, including their use in single-photon emitters, nano-flash memories^1,2^, photonic detectors^1,3^, and their role as qubits for spintronic quantum computing^1^. Moreover, QRs serve as a rich subject for exploring topological effects in condensed matter physics^1^. These nanostructures are remarkable due to their non-simply connected topology, which gives rise to intriguing energy structures^4^. These structures differ from most other low-dimensional systems such as quantum dots, quantum wires, and quantum wells. QRs can be categorized into two primary types, one-dimensional (1D) rings with a constant radius^5–13^ and two-dimensional rings (2D) with a variable radius^14–20^. Of particular note, a special case within the 1D QR family has gained significant recognition in the literature, known as the Aharonov-Bohm (AB) rings^21–24^. Currently, there are numerous works that delve into the properties of the AB rings, employing both theoretical and experimental approaches. For instance, AB rings have been studied in connection with the Aharonov-Casher effect^21–25^, violations of Lorentz symmetry^23^, mesoscopic decoherence^26^, electromagnetic resonators^27^, and the influence of Rashba spin-orbit coupling^24,28,29^.

In the framework of geometric theory of defects^30,31^, elastic deformations induced by topological defects in continuous media are described using a metric. These theory bears a resemblance to the theory of three-dimensional gravity. Within this geometric formalism, the continuous elastic medium is represented as a Riemann–Cartan manifold, where the curvature and torsion are associated with disclinations and dislocations, respectively, present in the medium. Consequently, the Burgers vector and Frank angle are respectively analogous to torsion and curvature. The impact of topological defects on the quantum dynamics of electrons/holes in a crystal has been explored in various physical scenarios^32,33^. Theoretical descriptions of quantum dynamics within a medium containing dislocations have been undertaken for quite some time. For instance, Kawamura^34^ and Bausch et al.^35–37^ investigated the scattering of a single particle within dislocated media using a distinct approach. They demonstrated that the equation describing the scattering of a quantum particle by a screw dislocation follows the Aharonov–Bohm form^38^. The Aharonov–Bohm effect has also been explored using the Katanaev–Volovich approach in media with a dislocation, as seen in Refs.^39,40^, and in the presence of dislocations, as observed in Refs.^33,41^. In Ref.^42^, Aurell probed deeper into the influence of dislocations on the properties of quantum dots. More recently, the study of the impact of topological defects in mesoscopic systems has been conducted in Ref.^43^, particularly with regard to a quantum dot in presence of a dislocation. Additionally, the emergence of spintronics has sparked interest in topological defects on the dynamics of spinful carriers. The interaction of the topological defect with the carriers in a spin-orbit coupled environment leads to impact on the spin dynamics, and thus could aid in designing spintronic devices through dislocation engineering. Although spintronic research has predominantly focused on defect-free materials, however, defects, due to their ability to couple with the magnetic moments could prove their utility in design of spintronic devices. Furthermore, curvature, which has been proposed as a tool for enhancing electronic nanodevice design, complements the potential of defects in this regard^44^.

A myriad of physical phenomena is induced by dislocations in real experiments. For instance, the time-reversal symmetry-breaking superconducting state along the (110) lattice direction in Sr_2_RuO_4_^45^, the influence of screw dislocations on the mechanical response of complex layered materials^46,47^, etc. Further, the interplay between real-space topological lattice defects and the reciprocal space topology of energy bands, may result in one-dimensional topological modes bound to screw dislocations in three-dimensional topological insulators. Dislocation-induced helical modes^48^, geometry-induced charge separation on a helicoidal ribbon^49^, and other phenomena further highlight the diverse effects produced by dislocations.

Moreover, recent studies on position-dependent mass Schrödinger particles in space-like screw dislocations^50^, as well as the application of quantum dots with screw dislocations in nonlinear optical^51^ and magneto-optical specifications^52^, have garnered significant attention in the scientific community.

Undoubtedly, one of the most promising materials of the century, graphene^53–56^, has attracted substantial attention in both theoretical and experimental investigations of QR systems. This heightened interest primarily arises from its remarkable properties. These include the involvement of linearly dispersive ‘massless’ Dirac fermions^57,58^, the potential emergence of topological phases resulting due to the violation of time reversal symmetry^59^, and the manifestation of Aharonov–Bohm oscillations in the presence of a magnetic field^60–64^, among others. Recently, numerous studies have been carried out to unravel the microscopic intricacies of graphene QRs under external magnetic fields, both with and without the introduction of spin-orbit couplings^63,65–76^. These investigations have illuminated the potential applications of graphene QRs in future optoelectronic^77^ and interferometric devices^78^.

In recent years, the $\alpha$-$T_3$ system has garnered considerable interest. By adjusting the parameter $\alpha$ in the range of [0:1], the $\alpha$-$T_3$ lattice offers a smooth transition between the honeycomb structure of graphene ($\alpha =0$) and the dice lattice ($\alpha =1$)^79–82^. This system can be experimentally realized in heterostructures and optical lattice setups, as previously proposed in various studies^83–85^. A nearest-neighbour tight-binding analysis reveals that the $\alpha$-$T_3$ lattice accommodates massless quasiparticles that obey the Dirac-Weyl equation, characterized by a generalized pseudospin dependent on the parameter $\alpha$. Numerous investigations have been carried out in recent years to explore a wide array of equilibrium and nonequilibrium properties of the $\alpha$-$T_3$ lattice^86–104^. On another front, topological phases in the Haldane dice lattice model^102,112^ and the Rashba dice model^83,113,114^ have attracted significant attention in recent years. Additionally, the observation of a quantum spin Hall phase transition in the $\alpha$-$T_3$ lattice has been noted^103^.

Driven by the promising potential of QRs, this study focuses onto the impact of a topological defect positioned at the center of the $\alpha$-$T_3$ QR, in presence of a Rashba spin-orbit coupling (RSOC). The interplay of RSOC and topological defect on the energy spectra and transport will comprise of the important discussions made in our paper. The introduction of this topological defect is achieved through a geometric description. Furthermore, our analysis involves into the dynamics of a charged particle constrained to navigate a QR with a fixed radius, all while being influenced by the presence of this topological defect. Additionally, we explore the Rashba SOC term, which adheres to the symmetries of the parent $\alpha$-$T_3$ ring. Notably, this term can be manipulated using an external electric field^115^, adding heavy adatoms etc., that effectively breaks the mirror symmetry with respect to the $\alpha$-$T_3$ plane. This discussion underscores the necessity for a comprehensive investigation into the behaviour of an $\alpha$-$T_3$ QR with Rashba SOC and topological defect. To further elucidate the controllability of persistent currents, we incorporate an external magnetic field into our analysis. These explorations have sparked substantial research activity, particularly in the context of potential applications within the emerging field of spintronics. An intriguing avenue for further investigation involves the combination of Rashba SOC with $\alpha$-$T_3$ to examine various properties. At the theoretical level, it is imperative to gain a deeper understanding of the cumulative effects of a robust electric field from SOC, confinement, and disorder potentials, such as the topological defect considered by us. These factors directly impact charge transport and the spin-related attributes of electrons.

This work is structured as follows. In “[Sec Sec2]”, we present the model incorporating a topological defect. “Results and discussions” provides an overview of the electronic properties of the system. The discussion of the effects of Burgers vector is included in “Current induced due to Burgers vector”. Finally, our findings are summarized in “Summary and conclusions”.

**Figure 1:**
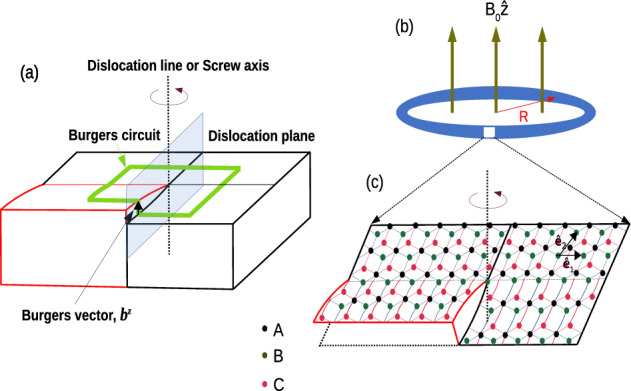
(**a**) Schematic diagram of a screw dislocation. Dislocation line, dislocation plane, Burgers circuit, and Burgers vector have shown in the figure. A counter-clockwise rotation is combined with a translation along the *z*-axis, resulting in a helical or spiral distortion. (**b**) A schematic diagram of the $\alpha$-$T_3$ ring of radius *R* subjected to a transverse magnetic field $B = B_0 {\hat{z}}$ and a screw dislocation. (**c**) Lattice structure of the $\alpha$-$T_3$ lattice is shown in the zoomed portion. Here, A, B, and C lattice sites are shown by black, blue, and red dots respectively. Hopping amplitude between the A and B sublattice is *t*, while between A and C is $\alpha t$. Black arrows labelled by $\hat{e_1}$ and $\hat{e_2}$ indicate the two translational vectors of the $\alpha$-$T_3$ lattice. The lattice is subjected to a screw dislocation.

## $\alpha$-$T_3$ AB ring with screw dislocation in addition with RSOC

In this section we shall investigate the dynamics of particles in a medium with a screw dislocation along the *z* direction as shown in Fig. 1. Before going to the details, let us briefly discuss the screw dislocation. A screw dislocation is a type of linear crystallographic defect or a dislocation within a crystal lattice. Dislocations cause disruptions in the regular arrangement of atoms in a crystal structure, and a screw dislocation is characterized by a helical or spiral distortion of the atomic planes around a central axis, known as the dislocation line. The dislocation line refers to the line along which the atomic arrangement in a crystal lattice is disrupted or distorted in the helical fashion. A dislocation plane is a theoretical surface or a plane that represents the boundary between two regions of a crystal lattice with different arrangements of atoms. In a screw dislocation, the dislocation plane is parallel to the dislocation line, the atomic planes on one side of the dislocation line are displaced relative to the planes on the other side. This displacement imparts a twisting or a screw-like distortion to the crystal lattice. Understanding the screw dislocation is crucial in analysing and predicting the behaviour of materials, especially in the context of mechanical properties, deformation, and plasticity. The Burgers vector, denoted as $b^z$ (see Fig. 1), represents the magnitude and direction of the lattice distortion associated with a dislocation. The Burgers circuit is a closed path around a dislocation line, and it is chosen in such a way that it encloses the dislocation and returns to the same lattice plane. The Burgers circuit provides information about the Burgers vector by examining the net translation of the crystal lattice around the dislocation. The displacement of the lattice planes along the Burgers circuit is equal to the Burgers vector.

The three-dimensional geometry of the medium, in this case, is characterized by non-trivial torsion which is identified with the surface density of the Burgers vector. Thus, the Burgers vector can be viewed as flux of torsion. The screw dislocation is described by the following metric, in cylindrical coordinates,

1 $$\begin{aligned} ds^2=g_{ij}dx^idy^j=d\rho ^2+(dz+\eta d\theta )^2+\rho ^2d\theta ^2, \end{aligned}$$

with $\rho >0$, $0\le \theta \le 2\pi$, and $-\infty \le z\le \infty$, and where $\eta$ is a parameter related to the Burgers vector $b^z$ by $\eta =b^z/2\pi$. A screw dislocation on a crystal surface is illustrated in Fig. 1. The illustration depicts a continuous warping of the surface as the growth front advances in the anti-clockwise direction. This process results in the generation of a helix with a specific pitch through accretion. The growth spirals (or helices) are readily observed and have been experimentally found in many crystals. They can be measured precisely by multiple beam interferometry^116,117^. In fact, several Burgers vectors may be found on a particular/single crystal surface.

Further, when we make a measurement of a physical vector quantity, however, we require the components of the vector in the original flat space (the laboratory coordinates). For example, the expectation value of the momentum is obtained by using the momentum operator,

2 $$\begin{aligned} \begin{aligned} \hat{\textbf{p}}=-i\hbar \nabla =-i\hbar \hat{e_i}\zeta ^{ij}\partial _j=-i\hbar \hat{e_i}\partial ^i,\\ \langle p^i \rangle = \langle \psi |{\hat{p}}^i|\psi \rangle , i=x,y,z, \end{aligned} \end{aligned}$$

where $\zeta ^{ij}=\delta ^{ij}$ is the flat-space metric, and $\psi$ is the wave function. If the wave function on the constrained surface is given, we transform the momentum operator as follows^118,119^,

3 $$\begin{aligned} {\hat{p}}^i=-i\hbar \partial ^i=-i\hbar g^{\mu \nu }\frac{\partial x^i}{\partial {\tilde{x}}^\mu }\frac{\partial }{\partial {\tilde{x}}^\nu }=-i\hbar \tilde{\partial ^\mu }x^i\tilde{\partial _\mu }. \end{aligned}$$

The coordinate indices in the curved space are given by Greek letters, and the curved space coordinates are denoted by ${\tilde{x}}^\mu$, with $\tilde{\partial _\mu }$ being the covariant derivative in the curved space. Concisely, the momentum operators for the metric given in Eq. (1) can be written in vector form as,

4 $$\begin{aligned} \begin{aligned} p^x&= -i\hbar \Big [\cos \theta \frac{\partial }{\partial \rho }-\frac{\sin \theta }{\rho }\frac{\partial }{\partial \theta }+\frac{\eta \sin \theta }{\rho }\frac{\partial }{\partial z} \Big ],\\ p^y&= -i\hbar \Big [\sin \theta \frac{\partial }{\partial \rho }+\frac{\cos \theta }{\rho }\frac{\partial }{\partial \theta }-\frac{\eta \cos \theta }{\rho }\frac{\partial }{\partial z} \Big ],\\ p^z&= -i\hbar \Big [\frac{\eta ^2+\rho ^2}{\rho ^2}\frac{\partial }{\partial z}-\frac{\eta }{\rho ^2}\frac{\partial }{\partial \theta } \Big ]. \end{aligned} \end{aligned}$$

We shall use these momenta to study the Rashba spin-orbit coupled Aharonov–Bohm $\alpha$-$T_3$ ring in presence of a topological defect.

Now, we consider the $\alpha$-$T_3$ quantum ring system includes Rashba SOC. The corresponding Hamiltonian can be written as, $H=H_0+H_R$, where $H_0$ is the tight-binding term, and $H_R$ is the Rashba spin-orbit coupling term. We write the Hamiltonian as^120^,

5 $$\begin{aligned} \begin{aligned} H&=-t\sum _{\langle ij\rangle \sigma }c_{i\sigma }^\dagger c_{j\sigma }-\alpha t\sum _{\langle ik\rangle \sigma }c_{i\sigma }^\dagger c_{k\sigma }-i\lambda _R\sum _{\langle ij\rangle \sigma \sigma ^\prime }c_{i\sigma }^\dagger ({\hat{D}}_{ij}\cdot \vec {\tau })_{\sigma \sigma ^\prime }c_{j\sigma ^\prime }-i\alpha \lambda _R\sum _{\langle ik\rangle \sigma \sigma ^\prime }c_{i\sigma }^\dagger ({\hat{D}}_{ik}\cdot \vec {\tau })_{\sigma \sigma ^\prime }c_{k\sigma ^\prime }+\bigg (h.c.\bigg ), \end{aligned} \end{aligned}$$

where $\sigma = \uparrow , \downarrow$, spin indices and *i*, *j*, *k* are labels for the sites corresponding to A, B, and C sublattices respectively. The first term is the electron hopping between the A and B sites, while the second one is that between the A and C sites. The summation of $\langle ij \rangle$ ($\langle ik \rangle$) runs over the nearest neighbour sites of A–B (A–C). Further, the Rashba SOC induced by electric fields due to a gradient of the crystal potential^83,113,114^, where $\vec {\tau }=(\tau _x,\tau _y,\tau _z)$ is the Pauli matrix vector, ${\hat{D}}_{ij}$ (${\hat{D}}_{ik}$) is the unit vector along the direction of the cross product $\vec {E}_{ij} \times \vec {r}_{ij}$ ($\vec {E}_{ik} \times \vec {r}_{ik}$) of the electric field $\vec {E}_{ij}$ ($\vec {E}_{ik}$) and displacement $\vec {r}_{ij}$ ($\vec {r}_{ik}$) for the bond *ij* (*ik*). $\lambda _R$ is the strength of Rashba SOC between the A and the B sites, while $\alpha \lambda _R$ is that between the A and the C sites. In momentum space, the Hamiltonian of the $\alpha$-$T_3$ lattice becomes,

6 $$\begin{aligned} H=\begin{pmatrix} 0 &{} -t\gamma _\text{k}^* &{} 0 &{} 0 &{} -i\lambda _R\gamma _{\text{k}+}^* &{} 0\\ -t\gamma _\text{k} &{} 0 &{} -\alpha t\gamma _\text{k}^* &{} i\lambda _R\gamma _{\text{k}-} &{} 0 &{} i\alpha \lambda _R\gamma _{\text{k}+}^*\\ 0 &{} -\alpha t\gamma _\text{k} &{} 0 &{} 0 &{} -i\alpha \lambda _R\gamma _{\text{k}-} &{} 0\\ 0 &{} -i\lambda _R\gamma _{\text{k}-}^* &{} 0 &{} 0 &{} -t\gamma _\text{k}^* &{} 0\\ i\lambda _R\gamma _{\text{k}+} &{} 0 &{} i\alpha \lambda _R\gamma _{\text{k}-}^* &{} -t\gamma _\text{k} &{} 0 &{} -\alpha t\gamma _\text{k}^*\\ 0 &{} -i\alpha \lambda _R\gamma _{\text{k}+} &{} 0 &{} 0 &{} -\alpha t\gamma _\text{k} &{} 0 \end{pmatrix} \end{aligned}$$

we defined $\gamma _\text{k}=1+e^{ik_1}+e^{ik_2}$ and $\gamma _{\text{k}\pm }=1+e^{i(k_1\pm 2\pi /3)}+e^{i(k_2\pm 4\pi /3)}$, where the components are along the axes indicated in Fig. 1c as $k_i=\vec {k}\cdot \hat{\text{e}}_i$. Our basis is $(c_{1\text{k}\uparrow },c_{2\text{k}\uparrow },c_{3\text{k}\uparrow },c_{1\text{k}\downarrow },c_{2\text{k}\downarrow },c_{3\text{k}\downarrow })$.

In the vicinity of a Dirac point (namely, **K**), and taking the momentum correction (4) due to screw dislocation into account, the Hamiltonian (6) corresponding to an ideal $\alpha$-$T_3$ ring is given by^6,73,74,121–125^,

7 $$\begin{aligned} H_{ring}=\frac{\hbar v_F}{R}\begin{pmatrix} 0 &{} -i(m-k\eta +\frac{1}{2})\cos \xi e^{\frac{i\pi }{3}} &{} 0 &{} 0 &{} -\frac{\lambda _R}{t}(m-k\eta +\frac{1}{2})\cos \xi e^{\frac{i\pi }{3}} &{} 0\\ i(m-k\eta +\frac{1}{2})\cos \xi e^{-\frac{i\pi }{3}} &{} 0 &{} -i(m-k\eta -\frac{1}{2})\sin \xi e^{\frac{i\pi }{3}} &{} \frac{\lambda _R}{t}(m-k\eta -\frac{1}{2})\cos \xi e^{\frac{i\pi }{3}} &{} 0 &{} -\frac{\lambda _R}{t}(m-k\eta +\frac{1}{2})\sin \xi e^{-\frac{i\pi }{3}}\\ 0 &{} i(m-k\eta -\frac{1}{2})\sin \xi e^{-\frac{i\pi }{3}} &{} 0 &{} 0 &{} -\frac{\lambda _R}{t}(m-k\eta -\frac{1}{2})\sin \xi e^{-\frac{i\pi }{3}} &{} 0\\ 0 &{} \frac{\lambda _R}{t}(m-k\eta -\frac{1}{2})\cos \xi e^{-\frac{i\pi }{3}} &{} 0 &{} 0 &{} i(m-k\eta -\frac{1}{2})\cos \xi e^{-\frac{i\pi }{3}} &{} 0\\ -\frac{\lambda _R}{t}(m-k\eta +\frac{1}{2})\cos \xi e^{-\frac{i\pi }{3}} &{} 0 &{} \frac{\lambda _R}{t}(m-k\eta -\frac{1}{2})\sin \xi e^{\frac{i\pi }{3}} &{} -i(m-k\eta -\frac{1}{2})\cos \xi e^{\frac{i\pi }{3}} &{} 0 &{} i(m-k\eta +\frac{1}{2})\sin \phi e^{-\frac{i\pi }{3}}\\ 0 &{} -\frac{\lambda _R}{t}(m-k\eta +\frac{1}{2})\sin \xi e^{\frac{i\pi }{3}} &{} 0 &{} 0 &{} -i(m-k\eta +\frac{1}{2})\sin \xi e^{\frac{i\pi }{3}} &{} 0 \end{pmatrix} \end{aligned}$$

where $\tan \xi =\alpha$ and $\hbar v_F=3at/2\cos \xi$. The eigenstates of the ring Hamiltonian can be obtained as,

8 $$\begin{aligned} \psi (R,\theta )=\begin{pmatrix} \chi _{1\uparrow }(R)e^{i\theta }\\ \chi _{2\uparrow }(R)\\ \chi _{3\uparrow }(R)e^{-i\theta }\\ \chi _{1\downarrow }(R)e^{-i\theta }\\ \chi _{2\downarrow }(R)\\ \chi _{3\downarrow }(R)e^{i\theta } \end{pmatrix}e^{im\theta }e^{ikz} \end{aligned}$$

where the integer *m* labels the orbital angular momentum quantum number, *k* is the momentum along the *z* direction and $\chi _i$’s denote the amplitudes corresponding to the three sublattices. Here, we investigate the electronic behaviour at a given value of radius $\rho$, namely $\rho = R$, such that the radial part is rendered frozen in the eigensolutions. For the sake of the hermiticity of the Hamiltonian in ring geometry, we made the replacements $\rho \rightarrow R$ and $\frac{\partial }{\partial \rho } \rightarrow -\frac{1}{2R}$ and obtain the energy spectrum as,

9 $$\begin{aligned} \begin{aligned} E_{1}&= 0\\ E_{2}&= \kappa \frac{\epsilon }{2}\Bigg \{\bigg [1+4\big (m-k\eta \big )^2-4\big (m-k\eta \big )\frac{1-\alpha ^2}{1+\alpha ^2}\bigg ]\bigg (1+\frac{\lambda _R^2}{t^2}\bigg )\Bigg \}^\frac{1}{2}\\ E_{3}&= \kappa \frac{\epsilon }{2}\Bigg \{\Big [1+4\big (m-k\eta \big )^2\Big ]\bigg (1+\frac{\lambda _R^2}{t^2}\frac{1-\alpha ^2}{1+\alpha ^2}\bigg )+ 4\big (m-k\eta \big )\bigg (\frac{\lambda _R^2}{t^2}+\frac{1-\alpha ^2}{1+\alpha ^2}\bigg )\Bigg \}^\frac{1}{2} \end{aligned} \end{aligned}$$

where $\kappa =\pm 1$ is the particle-hole index and $\epsilon =\frac{\hbar v_F}{R}$. $E_1$ is the zero energy flat band, $E_2$ and $E_3$ correspond to the energies for the $\uparrow$-spin and $\downarrow$-spin bands respectively. The energy spectra at the **K**-valley in presence of Rashba SOC and screw dislocation are expressed in Eq. (9). Further, the scattering effects due to the presence of screw dislocation are included as follows. In fact, the phase shift suffered by a particle incoming with a momentum *k* and scattered with angular momentum quantum number *m* by a screw dislocation may be derived as follows. The detailed calculations are provided in Ref.^126^. The resulting phase shifts can be expressed as^127–129^,

10 $$\begin{aligned} \begin{aligned} \delta _m(\vec {k})&= -\frac{\pi }{2}\bigg (|m+\frac{\vec {k}\cdot \vec {b^z}}{2\pi }|-|m|\bigg )= -\frac{\pi }{2}\bigg (|m+k\eta |-|m|\bigg ) \end{aligned} \end{aligned}$$

where $\eta$ is a parameter related to the screw dislocation, namely, $\eta =\frac{|\vec {b^z}|}{2\pi }$. This alteration in phase, induced by the presence of the screw dislocation, will have implications for the transport properties of the system. A detailed discussion on such effects will be presented in the subsequent sections.

## Results and discussions

### No magnetic field

**Figure 2:**
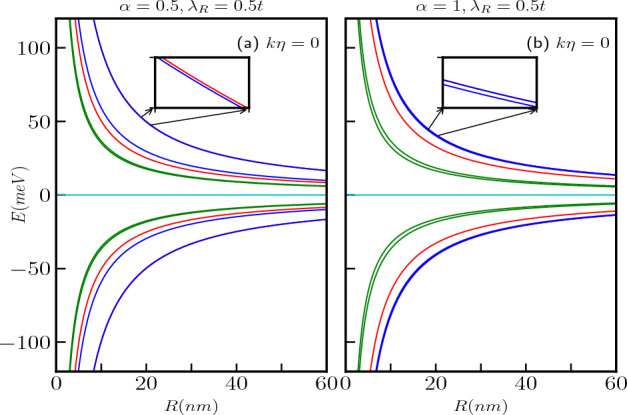
The spin-split energy spectra in the absence of a magnetic field for the $\alpha$-$T_3$ AB ring are depicted as a function of the ring radius, *R*, for quantum numbers $m=0$ (green curves), $m=1$ (blue curves), and $m=-1$ (red curves) in absence of a topological defect are presented in panels (a) for $\alpha =0.5$ and (b) for $\alpha =1$. The Rashba coupling parameter is all the while set at $\lambda _R=0.5t$. In the inset, we show the zoomed version for the same.

#### Without screw dislocation ($k\eta =0$)

We exclude the effects of topological defect, or what we call as screw dislocation ($k\eta =0$) to begin with. Figure 2a,b display the energies as a function of the ring radius, *R*, for various values of $\alpha$ at a fixed $\lambda _R$ value. One can easily verify the results of the $\alpha$-$T_3$ quantum ring without the RSOC term^125^ by setting $\lambda _R = 0$ ($k\eta =0$ anyway) in Eq. (9). In this case, we have considered a particular value for the RSOC, namely, $\lambda _R = 0.5t$ corresponding to $\alpha =0.5$ and 1, and plotted only the $m = -1$, 0, and 1 bands represented by red, green, and blue curves, respectively. When $\lambda _R=0$, the system exhibits three bands, with one being a flat band. However, with a non-zero $\lambda _R$ the original three bands split into six spin dependent bands, including two non-dispersive flat bands and four dispersive bands as described by Eq. (9). From Fig. 2, it is evident that all the energy branches have a 1/*R* dependence and approach $E\rightarrow 0$ for very large radii, irrespective of the value of $\alpha$. Additionally, the dispersive bands remain non-degenerate, in contrast to the case of the pseudospin-1 $\alpha$-$T_3$ QR without SOC. Moreover, the dispersive $\uparrow$-spin and $\downarrow$-spin bands split as well. Specifically, for $m=0$, the energies are given by,

11 $$\begin{aligned} E_2=\frac{\kappa \epsilon }{2}\sqrt{1+\frac{\lambda _R^2}{t^2}}, \end{aligned}$$

and

12 $$\begin{aligned} E_{3}=\frac{\kappa \epsilon }{2}\sqrt{1+\frac{\lambda _R^2}{t^2}\frac{1-\alpha ^2}{1+\alpha ^2}}. \end{aligned}$$

It can be observed that the $\uparrow$-spin energy band, $E_{2}$ is independent of $\alpha$, while the $\downarrow$-spin band, $E_{3}$ has a dependency on $\alpha$. This is an interesting result, since the $\uparrow$-spin band energies are insensitive to whether we are talking about graphene or the dice lattice. Consequently, the splitting between the $m=0$ bands (green curves in Fig. 2) increases with increasing values of $\alpha$. Whereas, the splitting between the bands with $m=-1$ (red curves in Fig. 2) and $m=1$ (blue curves in Fig. 2) decreases as $\alpha$ increases. Furthermore, the energy splitting decreases with increase of |*m*| values for all values of $\alpha$. In addition to that, the energy splitting increases with an increase of $\lambda _R$. An intriguing observation is that for $\alpha =1$, i.e., the dice lattice, the energies are given by,

13 $$\begin{aligned} E_{2}=\frac{\kappa \epsilon }{2}\sqrt{(1+4m^2)(1+\frac{\lambda _R^2}{t^2})}, \end{aligned}$$

and

14 $$\begin{aligned} E_{3}=\frac{\kappa \epsilon }{2}\sqrt{1+4m^2+4m\frac{\lambda _R^2}{t^2}}. \end{aligned}$$

Thus, $E_{2}$ is a even function of *m*, making it two-fold degenerate corresponding to $m=\pm 1,\pm 2, \pm 3, \ldots$ etc. On the other hand, the $E_{3}$ band is an odd function of *m*, resulting in it being non-degenerate as illustrated in Fig. 2b.

**Figure 3:**
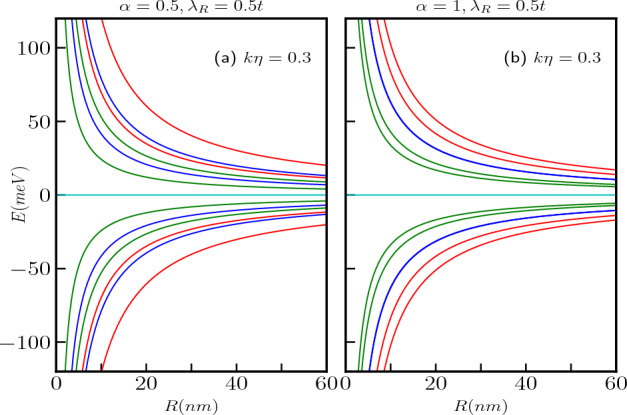
The spin-split energy spectra in the absence of a magnetic field for the $\alpha$-$T_3$ AB ring are depicted as a function of the ring radius, *R*, for quantum numbers $m=0$ (green curves), $m=1$ (blue curves), and $m=-1$ (red curves) in the presence of a topological defect with a magnitude of $k\eta =0.3$ are presented in panels (**a**) for $\alpha =0.5$ and (**b**) for $\alpha =1$. The Rashba coupling parameter is all the while set at $\lambda _R=0.5t$.

**Figure 4:**
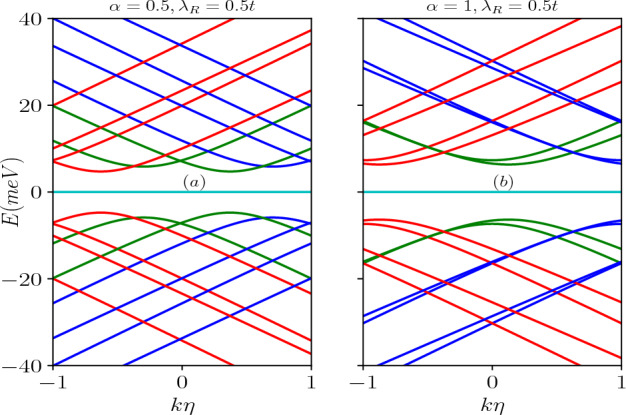
Energy levels in the absence of a magnetic field, as a function of the screw dislocation parameter $k\eta$, are depicted for (**a**) $\alpha =0.5$ and (**b**) $\alpha =1$, with a fixed ring radius of $R=50$ *nm*. The energy levels corresponding to different total angular momentum quantum numbers, namely $m=-1,-2$ (red curves), $m=0$ (green curves), and $m=1,2$ (blue curves), are presented. The Rashba coupling parameter remains constant at $\lambda _R=0.5t$.

#### With screw dislocation ($k\eta \ne 0$)

The scenario involving a topological defect arises when we implement the transformation via $m^\prime \rightarrow (m-k\eta )$ in the results of the Euclidean model. Consequently, upon examining Eq. (9), it becomes apparent that, unlike the situation where $k\eta =0$, all the energy levels for both $\uparrow$-spin and $\downarrow$-spin states, including the $m = 0$ bands, become dependent on the parameters $\alpha$ and $\lambda _R$. Hence, the splitting between the spin-split bands hinges on the presence of dislocations in the system, the parameter $\alpha$, the strength of the Rashba coupling, $\lambda _R$, as dictated by Eq. (9). Furthermore, for $\alpha = 1$, it is worth noting that $E_2$ and $E_3$ are non-degenerate, in contrast to the previous case. These findings are illustrated in Fig. 3a,b. Additionally, the quantum number *m* undergoes a shift that depends on $\eta$, which is associated with the Burgers vector. This shift is a manifestation of the Aharonov–Bohm effect, akin to what is observed in the context of a one-dimensional quantum ring penetrated by a magnetic flux^4^. The energy levels as a function of the strength of the screw dislocation are presented in Fig. 4 for two distinct cases, namely, $\alpha = 0.5$ and $\alpha = 1$ (dice lattice). Moreover, Eq. (9) makes it clear that the dispersive energy levels exhibit a hyperbolic dependence on the screw dislocation. It is important to note that the energy spectrum depends on the Burgers vector, $b^z=2\pi \eta$, as well as the radius, *R* of the ring, and hence associated with the geometry of the system.

**Figure 5:**
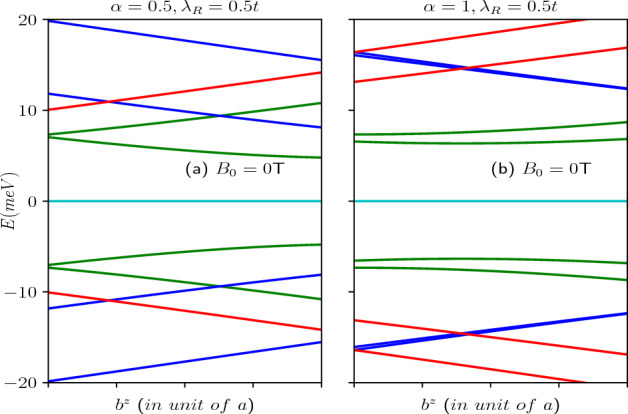
The energy spectra as a function of the Burgers vector in absence of magnetic field (**a**) for $\alpha =0.5$ and (**b**) for $\alpha =1$. The quantum number *m* ranges from $-1$ to 1. Positive values of *m* represented by blue curves, $m=0$ depicted by green curves, and negative values by red curves.

Additionally, there are energy extrema, with maxima in the valence bands and minima in the conduction bands, that pertain to different spin bands. The positions of these extrema are determined by the strength of dislocation as follows. For the $\uparrow$-spin bands, the extrema are found at

15 $$\begin{aligned} k\eta =m-\frac{1}{2}\frac{1-\alpha ^2}{1+\alpha ^2}, \end{aligned}$$

which is independent of $\lambda _R$. For the dice case ($\alpha =1$), the extrema occur at $k\eta =m$. However, for the $\downarrow$-spin bands, the extrema occurs at

16 $$\begin{aligned} k\eta =m+\frac{1}{2}\frac{\frac{\lambda _R^2}{t^2}+\frac{1-\alpha ^2}{1+\alpha ^2}}{1+\frac{\lambda _R^2}{t^2}\frac{1-\alpha ^2}{1+\alpha ^2}}. \end{aligned}$$

The expression above shows a dependency on $\lambda _R$ and hence we shall observe an interplay between the dislocation and RSOC. In fact, this interplay gives rise to interesting consequences as we shall see later. Furthermore, for each value of *m*, a band crossing point exists for $\alpha < 1$. However, in the case of $\alpha = 1$, no band crossings occur, instead, the bands touch each other at certain specific $k\eta$ values, depending upon the parameter $\lambda _R$.

The energy levels as a function of the Burgers vector ($b^z$) (in unit of the lattice constant) in the absence of a magnetic field are shown in Fig. 5a for $\alpha =0.5$ and (b) for $\alpha =1$. For a particular value of $\alpha$, increasing $b^z$ results in a linear increase in the conduction band energy levels corresponding to negative *m* values and a linear decrease in the energy levels for the positive values of *m*. Additionally, for $\alpha <1$ (here $\alpha =0.5$), the energy level of the $\uparrow$-spin in the $m=0$ band increases, while that for the $\downarrow$-spin level decreases. Moreover, when $\alpha =1$, the energy levels associated with the $m=0$ bands decrease as $b^z$ increases (shown in green curves in Fig. 5b). In the valence band, an opposite trend is observed where the energy levels for negative *m* decrease linearly, while the energy levels for positive *m* increase linearly. Furthermore, for $\alpha <1$, the $\downarrow$-spin energy level of the $m=0$ band increases, while that for the $\uparrow$-spin level decreases. With increase in $\alpha$, the energy difference between the spin-split bands diminishes (see Fig. 5a,b). However, it is worth noting that the $m=0$ bands remain as the low-energy bands in the absence of a magnetic field. The scenario in presence of the magnetic field is described later.

### Effect of magnetic field

**Figure 6:**
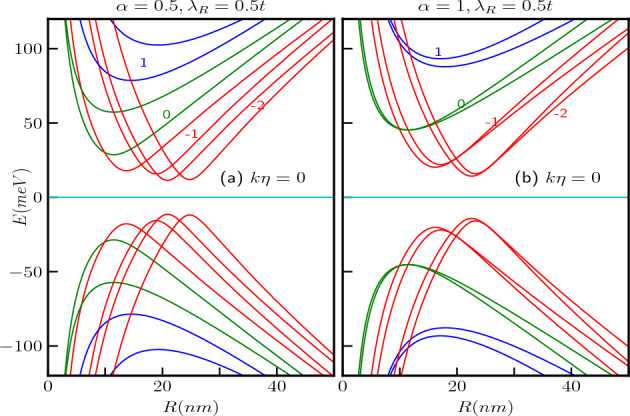
The energy spectra as a function of the ring radius *R* of the $\alpha$-$T_3$ quantum ring under the influence of an external magnetic field with a strength of $B_0=3$T at the $\textbf{K}$-valley are presented in panels (**a**) for $\alpha =0.5$ and (**b**) for $\alpha =1$, without topological defect. The parameters used in these calculations are $\lambda _R=0.5t$ and $t=1 eV$. The quantum number *m* ranges from $-2$ to 2, with positive values of *m* represented by blue curves, $m=0$ depicted by green curves, and negative values by red curves.

Now let us discuss the case when the $\alpha$-$T_3$ ring is threaded by a perpendicular magnetic field $\textbf{B}=B_0{\hat{z}}$. The only non-zero component of the vector potential is $A_\theta =B\rho /2$. Notice that, in the non-Euclidean metric of the dislocation, the vector potential that produces the uniform magnetic field, is identical to the flat space (Euclidean) potential vector. The spectrum of the system is modified by the field flux as follows,

17 $$\begin{aligned} \begin{aligned} E_{1}(\Phi )&= 0\\ E_{2}(\Phi )&= \kappa \frac{\epsilon }{2}\biggl \{\bigg [1+4\big (m-k\eta +\frac{\Phi }{\Phi _0}\big )^2-4\big (m-k\eta +\frac{\Phi }{\Phi _0}\big )\frac{1-\alpha ^2}{1+\alpha ^2}\bigg ]\bigg (1+\frac{\lambda _R^2}{t^2}\bigg )\biggl \}^\frac{1}{2}\\ E_{3}(\Phi )&= \kappa \frac{\epsilon }{2}\biggl \{\Big [1+4\big (m-k\eta +\frac{\Phi }{\Phi _0}\big )^2\Big ]\bigg (1+\frac{\lambda _R^2}{t^2}\frac{1-\alpha ^2}{1+\alpha ^2}\bigg )+ 4\big (m-k\eta +\frac{\Phi }{\Phi _0}\big )\bigg (\frac{\lambda _R^2}{t^2}+\frac{1-\alpha ^2}{1+\alpha ^2}\bigg )\biggl \}^\frac{1}{2} \end{aligned} \end{aligned}$$

where $\Phi =\pi R^2B_0$ is magnetic flux through the ring and $\Phi _0$ is the usual flux quantum ($=h/e$). The Zeeman coupling has been neglected at small enough values of the field. The addition of a magnetic field, represented by a U(1) minimal coupling with flux $\Phi$ threading the ring, breaks the time reversal symmetry allowing for the emergence of persistent charge currents^130^ which we shall discuss later.

#### Without screw dislocation ($k\eta = 0$)

In Fig. 6, we show the dependence of a few energy levels on the ring radius, *R*, considering $B_0 = 3$T for the two aforementioned cases i.e., $\alpha =0.5$ and 1 with $\lambda _R=0.5t$. Each level exhibits a non-monotonic behaviour as a function of the radius *R*. The energy levels attain an extremum (minimum for conduction band and maximum for valence band) at a particular value of *R*. However, the positions of these extrema depend on the values of *m*, $\alpha$ and $\lambda _R$ explicitly. In the limit of small *R*, all the energy levels vary inversely with *R*. On the other hand, the energy scales as, $E \sim |R|$ in limit of large *R*. Additionally, for a fixed magnetic field and for large *m*, the locations of the extrema points for different *m* depend on *R* as $R\propto \sqrt{|m|}$ irrespective of $\alpha$. Thus, the concept of large radii differs for different values of *m*. Consequently, for negative values of *m*, the extrema points of the energy exhibit a scaling behaviour, namely, $E_{min}\propto 1/\sqrt{|m|}$, resulting in a diminishing of the spectral gap with increasing |*m*|. Conversely, for positive values of *m*, the energy extrema scales as, $E_{min}\propto \sqrt{m}$. Furthermore, from Eq. (17), it is evident that in presence of a magnetic field, the energy splitting between the bands of the $m = 0$ level as well as the $m \ne 0$ levels decreases with the increase in the values of the parameters $\alpha$ and $\lambda _R$. Again, from Eq. (17) it is noted that for $\alpha < 1$, there are two points where the spin bands cross each other as a function of *R* for $m=0$ and negative values of *m*, i.e., $m=-1,-2,-3, \ldots$ etc. bands, whereas there is only one band crossing point for the positive values, namely, $m=1,2,3 \ldots$. These crossings obey the following condition,

18 $$\begin{aligned} \frac{\lambda _R^2}{t^2}\Big [1+4\big (m+\frac{\Phi }{\Phi _0}\big )^2-4\big (m+\frac{\Phi }{\Phi _0}\big )\Big ]-\frac{\lambda _R^2}{t^2}\frac{1-\alpha ^2}{1+\alpha ^2}\Big [1+4\big (m+\frac{\Phi }{\Phi _0}\big )^2+4\big (m+\frac{\Phi }{\Phi _0}\big )\Big ]-8\big (m+\frac{\Phi }{\Phi _0}\big )\frac{1-\alpha ^2}{1+\alpha ^2}=0. \end{aligned}$$

Equation (18) can be checked against the plot shown in Fig. 6a. Now, for the dice lattice case ($\alpha =1$), the above mentioned condition requires $m+\frac{\Phi }{\Phi _0}=\frac{1}{2}$, which implies that along the radius *R*, the energy band crossing points occur at $R=\sqrt{2}l_0\sqrt{(1/2-m)}$, where $l_0=\sqrt{\hbar /(eB_0)}$ is the magnetic length. Consequently, there is only one band crossing point for $m=0$ and $m=-1,-2,-3,\ldots$ etc. bands. Furthermore, band crossing is prohibited for positive values of *m* as there are no real values of *R* for $m>0$, indicating that the corresponding spin bands do not cross each other and hence there will not be any degeneracy as illustrated in Fig. 6b by the blue curves.

**Figure 7:**
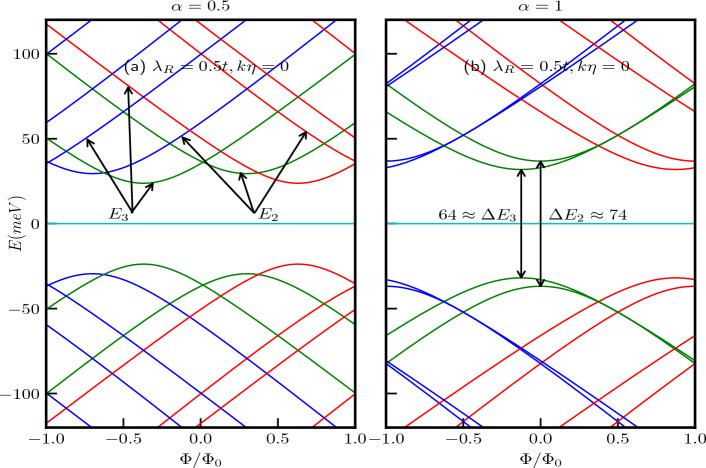
The energy levels in absence of screw dislocation ($k\eta =0$) as functions of the external magnetic flux $\Phi /\Phi _0$ with a fixed ring radius of $R=10$ nm, maintain the same conventions for the quantum number *m* as in the previous plots for (**a**) $\alpha =0.5$, $\lambda _R=0.5t$, and (**b**) $\alpha =1$, $\lambda _R=0.5t$.

The energy levels as a function of the external magnetic flux ($\Phi /\Phi _0$) are depicted in Fig. 7a,b for a quantum ring with $R=10$ *nm*, considering two cases, namely, $\alpha =0.5$ and $\alpha =1$ respectively with $\lambda _R=0.5$ in unit of *t*. The curves are represented by red, green, and blue colors corresponding to $m=-1$, $m=0$, and $m=1$, respectively. The magnetic field dependence of the energy spectra becomes evident when we rewrite Eq. (17) as,

19 $$\begin{aligned} E^2_{2}-\frac{\epsilon ^2}{4}\Big [1+4\big (m+\frac{\Phi }{\Phi _0}\big )^2-4\big (m+\frac{\Phi }{\Phi _0}\big )\frac{1-\alpha ^2}{1+\alpha ^2}\Big ]\Big (1+\frac{\lambda _R^2}{t^2}\Big )=0 \end{aligned}$$

and

20 $$\begin{aligned} \begin{aligned} E^2_{3}-\frac{\epsilon ^2}{4}\Big [\Big \{1+4\big (m+\frac{\Phi }{\Phi _0}\big )^2\Big \}\Big (1+\frac{\lambda _R^2}{t^2}\frac{1-\alpha ^2}{1+\alpha ^2}\Big )+4\big (m+\frac{\Phi }{\Phi _0}\big )\Big (\frac{\lambda _R^2}{t^2}+\frac{1-\alpha ^2}{1+\alpha ^2}\Big )\Big ]=0. \end{aligned} \end{aligned}$$

Thus, the energies display a hyperbolic dependence on the applied magnetic field, exhibiting extrema at the flux values given by,

21 $$\begin{aligned} \frac{\Phi }{\Phi _0}=-m+\frac{1}{2}\frac{1-\alpha ^2}{1+\alpha ^2} \end{aligned}$$

for $\uparrow$-spin band $E_{2}$, the extrema points are independent of the strength of the Rashba coupling, but depends on the values of *m* and the parameter $\alpha$. For the dice lattice ($\alpha =1$), the extrema occur at $\Phi /\Phi _0=-m$. However, the extrema for the $\downarrow$-spin band $E_{3}$ occur at

22 $$\begin{aligned} \frac{\Phi }{\Phi _0}=-m-\frac{1}{2}\frac{\frac{\lambda _R^2}{t^2}+\frac{1-\alpha ^2}{1+\alpha ^2}}{1+\frac{\lambda _R^2}{t^2}\frac{1-\alpha ^2}{1+\alpha ^2}}, \end{aligned}$$

showing a dependency on the strength of Rashba SOC, $\alpha$ and *m*. For the dice lattice, the extrema are obtained at $\Phi /\Phi _0=-m-\frac{1}{2}\frac{\lambda _R^2}{t^2}$. The energy gaps at the extrema points, that is, the minimum values of the gap are given by,

23 $$\begin{aligned} \begin{aligned} \Delta E_{2}&=\frac{2\epsilon \alpha }{1+\alpha ^2}\sqrt{1+\frac{\lambda _R^2}{t^2}},\\ \Delta E_{3}&=\frac{2\epsilon \alpha }{1+\alpha ^2}\sqrt{\frac{1-\frac{\lambda _R^4}{t^4}}{1+\frac{\lambda _R^2}{t^2}\frac{1-\alpha ^2}{1+\alpha ^2}}}. \end{aligned} \end{aligned}$$

Therefore, it is observed that for a fixed value of Rashba coupling, the energy gaps for both the spin bands increase with increase in $\alpha$. However, the minimum energy gap for both the spin bands is independent of *m*. Also the $\downarrow$-spin bands, namely, $E_{3}$ have lower energies than the $\uparrow$-spin bands ($E_2$). The $\uparrow$-spin $E_2$ and $\downarrow$-spin $E_3$ bands are illustrated in the Fig. 7a. For the dice lattice case, and for $\lambda _R=0.5t$, the energy gaps are obtained as, $\Delta E_{2}\approx 74$ meV and $\Delta E_{3}\approx 64$ meV which can be verified from the Fig. 7b. Furthermore, from Fig. 7 it is evident that $E_{2}(m)\ne E_{2}(-m)$ and $E_{3}(m)\ne E_{3}(-m)$, indicating the existence of finite spin currents.

**Figure 8:**
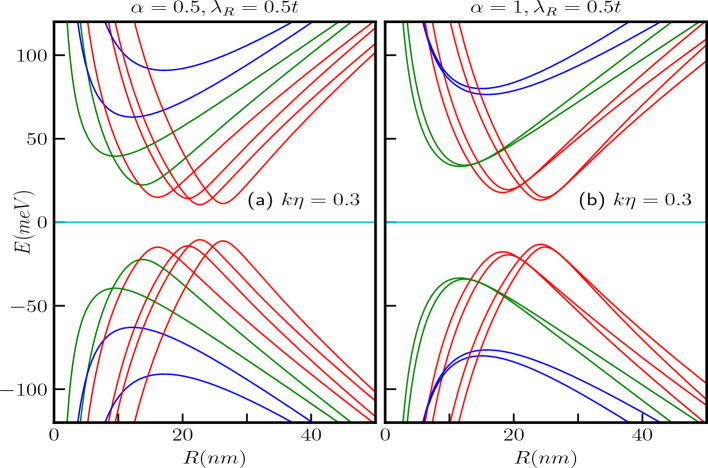
The energy spectra as a function of the ring radius *R* of the $\alpha$-$T_3$ quantum ring under the influence of an external magnetic field with a strength of $B_0=3$T at the $\textbf{K}$-valley are presented in panels (**a**) for $\alpha =0.5$ and (**b**) for $\alpha =1$, we introduce a screw dislocation with a magnitude of $k\eta =0.3$. The parameters used in these calculations are $\lambda _R=0.5t$ and $t=1$ eV. The quantum number *m* ranges from $-2$ to 2, with positive values of *m* represented by blue curves, $m=0$ depicted by green curves, and negative values by red curves.

#### With screw dislocation ($k\eta \ne 0$)

Let us now delve into the impact of a topological defect, specifically a screw dislocation, on the electronic spectra of the $\alpha$-$T_3$ QR. Referring to Eq. (17), we find that all of the previously discussed characteristics remain unaltered, except we may consider that *m* as an effective quantum number, let us call it as $m^\prime$ with $m^\prime \rightarrow (m - k\eta )$ which has a shift in the *m* values by an amount of $k\eta$. The results are visually represented in Fig. 8a,b, while considering $\alpha$ values of 0.5 and 1, with the parameter $k\eta$ set to a modest value, say $k\eta =0.3$. Again, for the case of $\alpha = 1$, the band crossing points emerge at $R=\sqrt{2}l_0\sqrt{\{1/2-(m-k\eta )\}}$, signifying a shift to the right as depicted in Fig. 8b.

**Figure 9:**
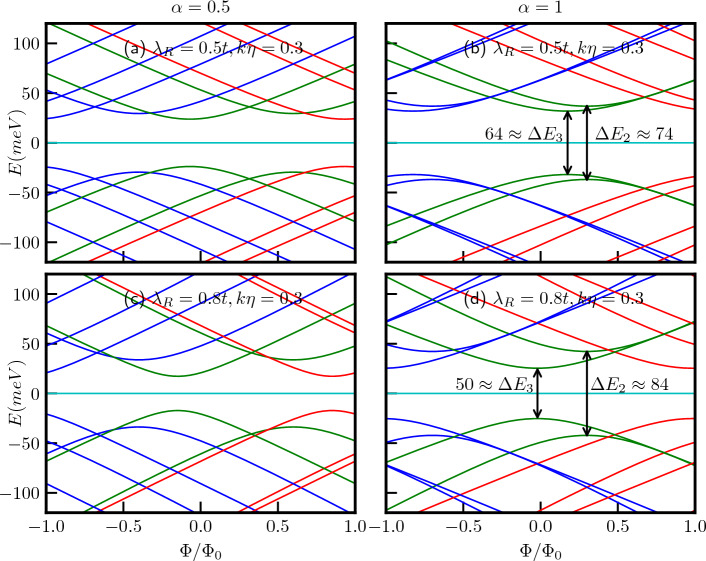
The energy levels, as functions of the external magnetic flux $\Phi /\Phi _0$ with a fixed ring radius of $R=10$ nm, maintain the same conventions for the quantum number *m* as in the previous plots. We explore various combinations of $\alpha$, $\lambda _R$, and $k\eta$ as follows: (**a**) $\alpha =0.5$, $\lambda _R=0.5t$, and $k\eta =0.3$, (**b**) $\alpha =1$, $\lambda _R=0.5t$, and $k\eta =0.3$, (**c**) $\alpha =0.5$, $\lambda _R=0.8t$, and $k\eta =0.3$, and (**d**) $\alpha =1$, $\lambda _R=0.8t$, and $k\eta =0.3$.

Furthermore, the conditions for the extremal points defined in Eqs. (21) and (22) are also modified due to $m^\prime \rightarrow (m - k\eta )$, resulting in a displacement of these extremal points by an amount equal to $k\eta$ in the positive direction, as illustrated in Fig. 9a–d. It is important to note that the energy gaps at these extremal points, as per Eq. 23, are not influenced by the presence of the topological defect ($k\eta$), their dependencies solely rely on the parameters $\alpha$ and $\lambda _R$. To demonstrate this, we have considered two values of $\lambda _R$, namely, $\lambda _R = 0.5t$ and $\lambda _R = 0.8t$. The energy gap values for the $\alpha = 1$ case are presented in the respective figures (see Fig. 9).

In this particular scenario, we are dealing with a one-dimensional quantum ring in the presence of a screw dislocation and Aharonov–Bohm flux. The energy spectrum displays a parabolic dependence on the Burgers vector, similar to its dependence on the magnetic flux. Upon analysing our findings, we can infer that a particle within a space featuring a topological defect behaves similarly to a particle in a Euclidean space in the presence of an effective magnetic flux traversing the ring. This effective flux is a combination of two contributions, the first is of a topological nature stemming from the topological defect, while the other is due to the magnetic flux $\Phi$. By adjusting the magnetic flux as an external fine-tuning parameter to counterbalance the topological contribution introduced by the defect, we can nullify the Aharonov-Bohm effect in the ring. In such cases, the energy spectrum resembles that of a particle moving in a quantum ring within a space devoid of any topological defect. This is a very important result. It provides a clue how the effects of a topological defect can be totally or partially compensated by an external field with regard to the observation of the AB effect. Further consequences on the spin and charge currents are elucidated below.

**Figure 10:**
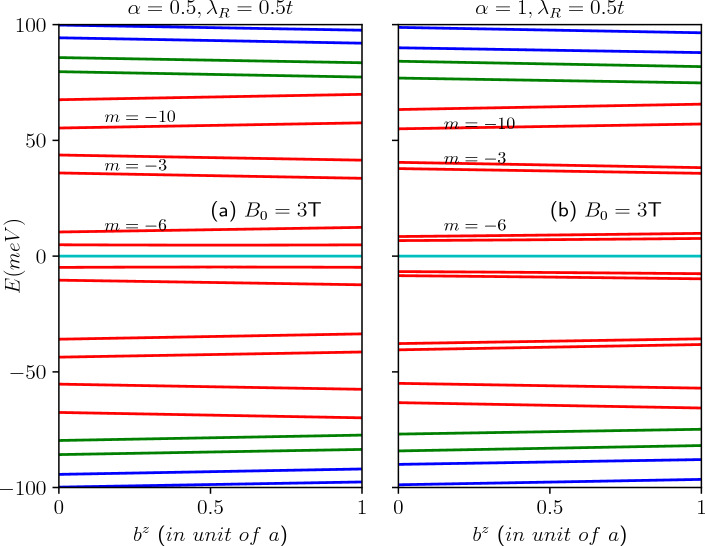
The energy spectra as a function of the Burgers vector in presence of magnetic field of $B_0=3$T (**a**) for $\alpha =0.5$ and (**b**) for $\alpha =1$. The quantum numbers are shown in the plots. Positive values of *m* represented by blue curves, $m=0$ depicted by green curves, and negative values by red curves.

The energy levels as a function of the Burgers vector in presence of a magnetic field of $B_0=3$T are illustrated in Fig. 10a for $\alpha =0.5$ and (b) for $\alpha =1$. The plots show a deviation from the previous scenario corresponding to zero magnetic field. In this case, the energy levels of both the conduction and the valence bands remain nearly unchanged as a function of $b^z$. Notably, the $m=0$ bands no longer represent the lowest energy states, instead, the negative *m* bands possess lower energy than the $m=0$ bands in presence of the magnetic field. These bands contribute significantly to the transport properties of the system. As the quantum number *m* becomes more negative, the energy difference between the valence and the conduction bands decreases. Specifically, for $m=-6$, the system exhibits the minimum spectral gap. Subsequently, the gap between the conduction bands and valence bands increase with |*m*|, as illustrated in Fig. 10a,b. Additionally, as $\alpha$ increases, the energy difference between the spin-split bands for negative *m* decreases, while that for the $m\ge 0$ bands increases. Thus, a screw dislocation, among other things, presents distinct spectral properties in presence and absence of an external magnetic field.

**Figure 11:**
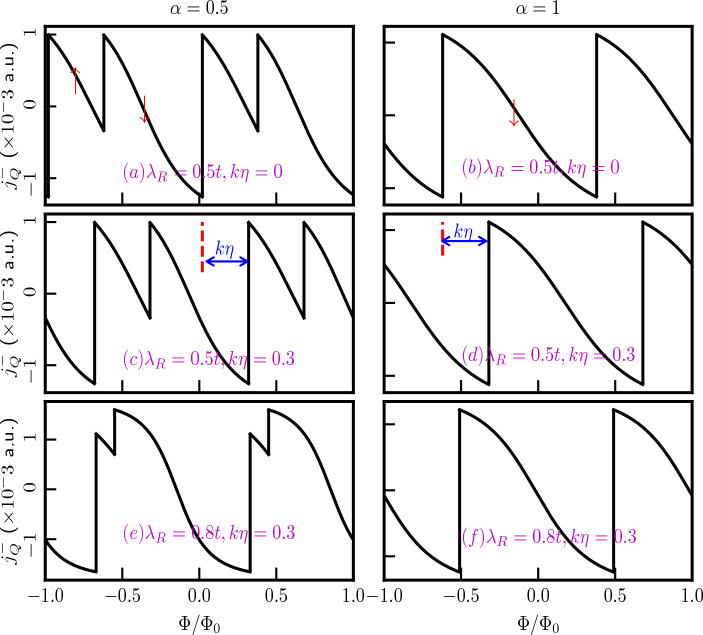
The charge persistent currents are plotted as functions of the external magnetic flux, considering the low-energy states without accounting for the topological defect, in panels (**a**) for $\alpha =0.5$ and (**b**) for $\alpha =1$, with $\lambda _R=0.5t$. In panels (**c**) and (**d**), the same currents are presented in the presence of the screw dislocation term $k\eta =0.3$, for $\alpha =0.5$ and $\alpha =1$ respectively. Panels (**e**) and (**f**) highlight the impact of the Rashba SOC as we increase the $\lambda _R$ parameter to 0.8*t*, while keeping $k\eta =0.3$ fixed for $\alpha =0.5$ and 1, respectively. In panels (**a**) and (**b**), the labels $\uparrow$ and $\downarrow$ denote the currents from the corresponding spin bands. In panels (**c**) and (**d**), the phase shift is represented by the blue arrows. Here, a.u. refers to arbitrary unit where we have considered $\hbar = e = v_F =1$.

### Charge persistent current

The charge persistent current in the low-energy state can be calculated using the linear response formula, $j_Q=-\sum _{m,\kappa }\frac{\partial E}{\partial \Phi }$, where the sum refers to all (and only) the occupied states (for the valence band ($\kappa =-1$)) and the *m* values are chosen carefully to perform the summation. Since the current is periodic in $\Phi /\Phi _0$ with a period of 1 (that is $\Phi =\Phi _0$), we restrict the discussion to the window $-1\le \Phi /\Phi _0 \le 1$. The analytical form for the charge current is obtained as,

24 $$\begin{aligned} \begin{aligned} j_{Q}^\kappa&=-\frac{\epsilon ^2\kappa }{2\Phi _0}\sum _m\frac{\big (1+\frac{\lambda _R^2}{t^2}\big )\Big [2\big (m-k\eta +\frac{\Phi }{\Phi _0}\big )-\frac{1-\alpha ^2}{1+\alpha ^2}\Big ]}{E_2(\Phi )}\\&-\frac{\epsilon ^2\kappa }{2\Phi _0}\sum _m\frac{2\big (m-k\eta +\frac{\Phi }{\Phi _0}\big )\big (1+\frac{\lambda _R^2}{t^2}\frac{1-\alpha ^2}{1+\alpha ^2}\big )+\big (\frac{\lambda _R^2}{t^2}+\frac{1-\alpha ^2}{1+\alpha ^2}\big )}{E_3(\Phi )}. \end{aligned} \end{aligned}$$

The spin branches closest to the Fermi energy exhibit non-monotonic behaviour, resulting in two distinct contributions to the charge current coming from the $\uparrow$-spin and $\downarrow$-spin components. Since we are calculating the current contributions arising from the low-energy states, it is clear from Fig. 7 that for a certain range of $\Phi /\Phi _0$, only one energy state labelled by a particular value of *m* is present. Hence, the sum in Eq. (24) comprises of only one value of *m*. Furthermore, based on the observations in Fig. 7, we can discern that the low-energy states when $\alpha = 0.5$ encompass both the spin bands. Consequently, in our current calculations, we considered contributions from both the spin branches. In contrast, for the $\alpha = 1$ scenario, the low-energy state exclusively comprises the $\downarrow$-spin branches, and thus, we only have accounted for the contributions from the $\downarrow$-spin to compute the persistent current. The outcomes of these calculations are depicted in Fig. 11 for both $\alpha = 0.5$ and $\alpha = 1$, considering various combinations of $\lambda _R$ and $k\eta$, all the while maintaining a fixed ring radius of $R = 10$ nm. The asymmetric spectral features between the two spin branches allows for the possibility of a net spin currents, as we shall see below. For all values of $\alpha$, the persistent currents oscillate periodically with $\Phi /\Phi _0$, with a periodicity of $\Phi /\Phi _0=1$. Figure 11a,b illustrate that the persistent currents can be tuned by adjusting the parameter $\alpha$ for a fixed value of the Rashba coupling ($\lambda _R$). Moreover, the charge persistent currents can be manipulated via $\lambda _R$ for a fixed $\alpha$, (see Fig. 11a,c,e) since the Rashba parameter can be controlled by a gate voltage. Further, it is worth noting the presence of a kink in the current profile when $\alpha < 1$. This kink arises because different spin bands contribute to the current, as indicated by the distinctions denoted by $\uparrow$ and $\downarrow$ for their respective spin bands. However, this kink phenomenon is absent for the dice lattice ($\alpha = 1$), as evident in Fig. 11b,d,f. The reason being, in this case, only the $\downarrow$-spin bands contribute to the current, as mentioned earlier.

Furthermore, the topological defect plays a pivotal role in the behaviour of the charge current. As illustrated in Fig. 11, we can observe that, for a fixed value of $\lambda _R$, the screw dislocation induces a phase shift in the current, regardless of the specific value of $\alpha$. For example, every point of the current profile is shifted by the same amount as the strength of the topological defect as shown in the middle panel of Fig. 11. However, the topological defect does not influence the current profile itself. A desired shift in the current profile may be achieved via the controllable parameter $k\eta$. Further, the Rashba coupling, $\lambda _R$ plays a role as well. The depth in the kink of the current profile decreases with the increasing $\lambda _R$ (see bottom panel of Fig. 11). This can be understood from the lower panel of Fig. 7, as the increase in $\lambda _R$ results in a decrease in the $\uparrow$-spin contribution in the low-energy state within a certain range of $\Phi /\Phi _0$. However, the overall oscillation period remains unaltered.

In summary, we can manipulate the persistent current profile by fine-tuning the Rashba coupling, and we have the ability to shift the phase of the current to suit our specific applications by adjusting the strength of the topological defect.

**Figure 12:**
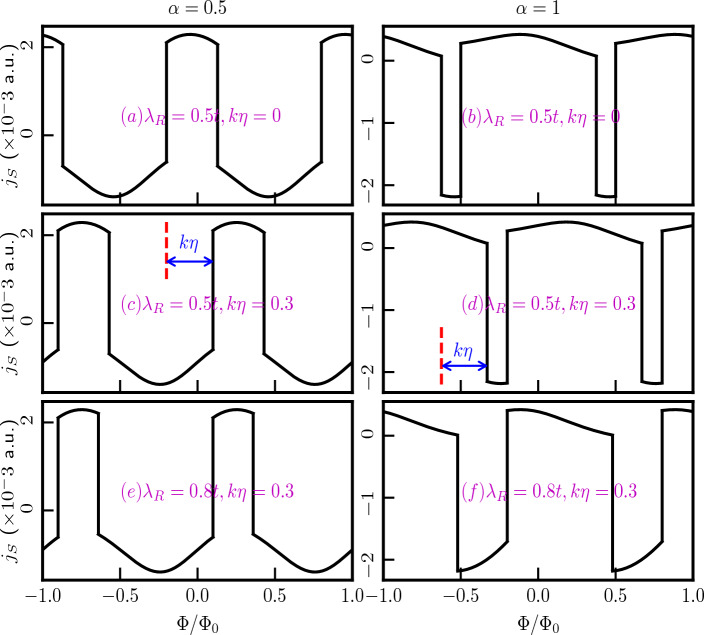
The equilibrium spin currents are plotted as functions of the external magnetic flux, considering the low-energy states without accounting for the topological defect, in panels (**a**) for $\alpha =0.5$ and (**b**) for $\alpha =1$, with $\lambda _R=0.5t$. In panels (**c**) and (**d**), the same currents are presented in the presence of the screw dislocation term $k\eta =0.3$, for $\alpha =0.5$ and $\alpha =1$, respectively. Panels (**e**) and (**f**) highlight the impact of the Rashba SOC as we increase the $\lambda _R$ parameter to 0.8*t*, while keeping $k\eta =0.3$ fixed for $\alpha =0.5$ and 1, respectively. In panels (**c**) and (**d**), the phase shift is represented by the blue arrows.

### Equilibrium spin currents

We shall now study equilibrium spin currents. In contrast to the formalism for obtaining the charge current, one can obtain the spin currents by accounting for distinct velocities for different spin branches. Thus, we define equilibrium spin current as,

25 $$\begin{aligned} j_S=j_Q(\uparrow )-j_Q(\downarrow ). \end{aligned}$$

We have calculated the equilibrium spin currents following the procedure discussed earlier. The peculiar separation of the spin branches results in differences of the velocities between the two spin projections, giving rise to a spin current, as shown in Fig. 12. The figure illustrates a significant spin current for small values of the flux, which can be attributed to the large charge current originating from a single spin branch.

The striking feature is that the magnitude as well as the pattern of the spin currents depend upon the parameters $\alpha$ and the strength of the Rashba coupling ($\lambda _R$). We present results for $\alpha =0.5$, and $\alpha =1$ with different values of $\lambda _R$ and topological defect $k\eta$ for a ring of radius, $R=10$ nm. The presence of the Rashba coupling breaks inversion symmetry (in addition to the $\sigma _z$ symmetry) in the plane even for small $\lambda _R$. The symmetry breaking determines the spin labelling of the energy branches that take part in yielding the spin currents. Additionally, the spin currents exhibit periodic behaviour with $\Phi /\Phi _0$, with a periodicity equal to one flux quantum, irrespective of $\alpha$. Similar to the case of charge persistent current, we also notice a phase shift introduced by the topological defect. The magnitude of this phase shift precisely corresponds to the strength of the defect.

**Figure 13:**
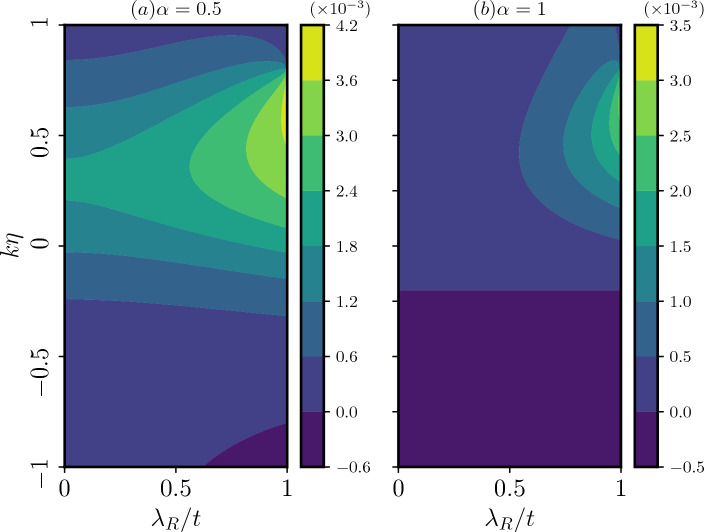
The equilibrium spin currents are plotted as functions of the strength of the Rashba SOC and the topological defect, considering a fixed magnetic flux, $\Phi /\Phi _0=0.3$, and angular momentum quantum number $m=0$, panels (**a**) for $\alpha =0.5$, and (**b**) for $\alpha =1$.

To gain further insights, we present a color plot illustrating the equilibrium spin current ($j_S$) as a function of the strength of Rashba SOC and the topological defect in Fig. 13. The color map allows us to observe the interplay between these two parameters at a specific value of the magnetic flux, namely, $\Phi /\Phi _0=0.3$. Further, we have considered the angular momentum quantum number fixed at $m=0$, which is most relevant for the flux used here. The observed behaviour reveals that the spin persistent current oscillates between negative and positive values as we tune the strength of the topological defect for a fixed value of $\lambda _R$. For $\alpha <1$ (see Fig. 13a), we observe a significant variation as a function of the defect in the spin persistent current for all values of $\lambda _R$ (in unit of *t*). In contrast, for $\alpha =1$ (as depicted in Fig. 13b), the variation is less pronounced for small values of $\lambda _R$, whereas it becomes more significant as we increase $\lambda _R$. Furthermore, the color map suggests that to achieve the maximum spin persistent current for a spintronic device, one requires a high value of $\lambda _R$ and the strength of the topological defect to have close to 0.5. The role played by $\lambda _R$ is known in literature, while that due to the topological defect and their interplay are less known. It is also worth noting that the results are presented for a value $\Phi /\Phi _0=0.3$, however the qualitative nature of the plot remains consistent for other magnetic flux values and angular momentum quantum numbers (*m*). In practical applications, by fine-tuning external parameters such as magnetic flux, dislocation, and the strength of the Rashba SOC, one can tailor the spin current to suit specific device requirements. However, we have to note that the qualitative characteristics of the plot remain consistent even if $\lambda _R/t>1$, that is, achieving maximum spin persistent current necessitates high values of $\lambda _R$ and the strength of the topological defect ($k\eta$). Further, the detailed behaviour of the plots may vary depending on the quantum number *m*. As we increase the values of $\lambda _R/t$, different *m* values start contributing to the spin current.

These findings underscore the potential of $\alpha$-$T_3$ quantum rings as key components for spintronic applications, where we can manipulate the performance of the devices by adjusting both the strength of the Rashba coupling and the topological defect.

### Dipole moment of the ring: applications to spintronics

**Figure 14:**
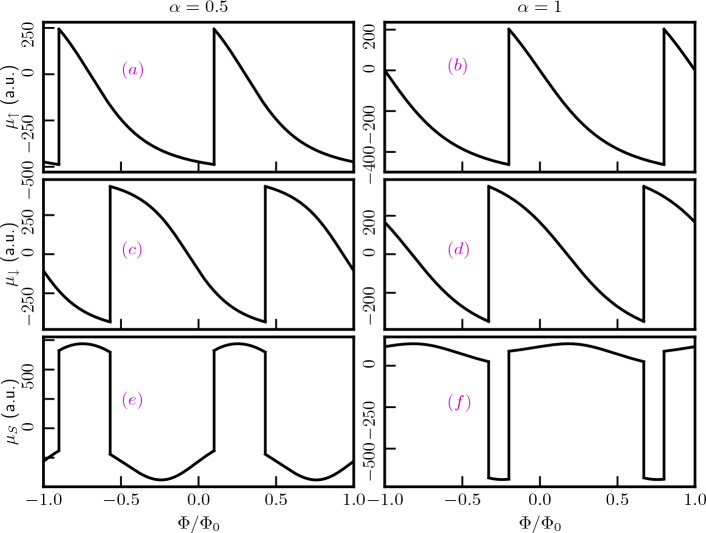
The induced dipole moments are plotted as function of the external magnetic flux, panels (**a**) for $\alpha =0.5$, $\uparrow$-spin current, (**b**) for $\alpha =1$, $\uparrow$-spin current, (**c**) for $\alpha =0.5$, $\downarrow$-spin current, (**d**) for $\alpha =1$, $\downarrow$-spin current, (**e**) for $\alpha =0.5$, total spin current, and (**f**) for $\alpha =1$, total spin current. The other parameters are taken as $\lambda _R=0.5t$ and $k\eta =0.3$.

Continuing with the aforementioned studies, we now can compute the induced dipole moment of the $\alpha$-$T_3$ ring. This is attributed to the persistent currents flowing in the charge and spin sectors circulating around the ring. By defining the induced dipole moment as the product of the charge current density ($j_Q$) or the spin current density ($j_S$) and the area of the quantum ring, denoted as $\mu$, we may get useful information. In Fig. 14, we illustrate the dipole moments for various scenarios, namely, $\uparrow$-spin charge current for $\alpha =0.5$ (Fig. 14a), $\uparrow$-spin charge current for $\alpha =1$ (Fig. 14b), $\downarrow$-spin charge current for $\alpha =0.5$ (Fig. 14c), and $\downarrow$-spin charge current for $\alpha =1$ (Fig. 14d). Additionally, in Fig. 14e,f, we display the total spin dipole moment for $\alpha =0.5$ and $\alpha =1$, respectively. The parameters $\lambda _R=0.5t$ and $k\eta =0.3$ are held constant throughout. It is apparent from Fig. 14 that the dipole moments originating from different spin branches are neither uniform nor equivalent, instead, they exhibit oscillatory behavior with a period of $\Phi _0$. Such charge and spin dipole moments have the prospects of inducing spin torque in the ring system, a key aspect in the realm of spintronic devices. Moreover, the ability to manipulate both the charge and spin persistent currents, along with the dipole moments, through parameters such as $\alpha$, $\Phi$, and $\lambda _R$, coupled with modulation by the degree of dislocation ($k\eta$), highlights the system’s controllability. This opens up avenues for tailored applications in spintronics.

## Current induced due to Burgers vector

In general the Hamiltonian of the system in a screw dislocated medium given in Eq. (1) can be represented in the form,

26 $$\begin{aligned} {\mathscr {H}}=\frac{1}{2m\sqrt{g}}\big (-i\hbar \partial _i - {\mathscr {A}}_i\big )\sqrt{g}g^{ij}\big (-i\hbar \partial _j - {\mathscr {A}}_j\big ) \end{aligned}$$

where ${\mathscr {A}_{\mu }}(\mu =0,x,y,z)$ denote the components of the gauge field. The non-Abelian potential ${\mathscr {A}}$ captures the effects (including the spin-orbit coupling) if one makes the following identifications where ${\mathscr {A}}_0=-\frac{e\hbar }{mc}B^a\tau ^a$ and ${\mathscr {A}}_{ind}$ is the screw dislocation induced vector potential (contained in the spatial coordinates *x*, *y*, *z*). $B^a$ is the external magnetic field and $\tau ^a=S^a/2$ are the generators of SU(2) corresponding to the spin-1 operator. The external magnetic field is in the *z*-direction. Further, even in absence of an external magnetic field, the screw dislocation effectively acts as an artificial gauge field that gives rise to a pseudo-magnetic field, $B_s=(\nabla \times {\mathscr {A}}_i)_z$ perpendicular to the $\alpha$-$T_3$ lattice plane.

**Figure 15:**
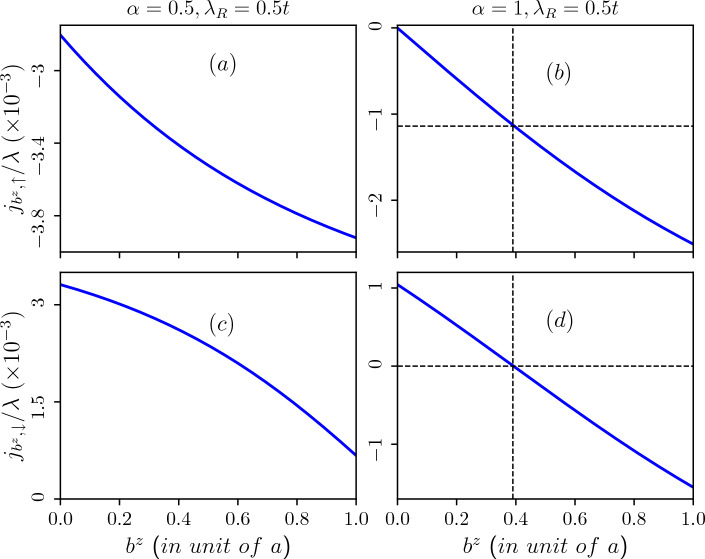
Burgers current as a function of the Burgers vector induced by the topological screw dislocation in absence of external magnetic field. Upper panel: corresponds to $\uparrow$-spin (**a**) for $\alpha =0.5$ and (**b**) for $\alpha =1$. Lower panel: corresponds to $\downarrow$-spin (**c**) for $\alpha =0.5$ and (**d**) for $\alpha =1$. The Rashba spin-orbit coupling parameter is taken as $\lambda _R=0.5t$.

Following the formalism of Refs.^131–134^, due to this effective field ${\mathscr {F}}_{i0}=\partial _i {\mathscr {A}}_0-\partial _t {\mathscr {A}}_i-i[{\mathscr {A}}_i,{\mathscr {A}}_0]$, there is a dissipative current $j_i^a$ which is conjugate to the effective field. Calculating the time derivative of the expectation value of ${\mathscr {H}}$ (given in Eq. (26)) we find that,

$$\begin{aligned} \frac{d}{dt}\langle {\mathscr {H}} \rangle = \int dr j_i^a {\mathscr {F}}_{i0}^a. \end{aligned}$$

Armed with the gauge invariant Hamiltonian and the variational definition of $j_i^a$, we are ready to calculate the equilibrium charge and spin currents induced by the screw dislocation. Following the same technique as earlier in the presence of an external magnetic field, one naturally expects an orbital response in the form of a diamagnetic current. This current is given by the derivative of the energy $E[{\mathscr {A}}_i^a](=\langle {\mathscr {H}}\rangle )$ with respect to ${\mathscr {A}}_i^a$. As $E[{\mathscr {A}}_i^a]$ is gauge invariant, it can depend only on the effective gauge field, namely,

27 $$\begin{aligned} {\mathscr {F}}_{ij}=\partial _i{\mathscr {A}}_j-\partial _j{\mathscr {A}}_i-i[{\mathscr {A}}_i,{\mathscr {A}}_j]. \end{aligned}$$

A particular form of the invariant is determined by the symmetry of a particular system. Firstly, we assume that the external magnetic field is zero, i.e., ${\mathscr {A}}_0=0$. Since in the absence of ${\mathscr {A}}_i^a$ the system is rotationally invariant, the first spin-orbit (SO) correction to the energy must be proportional to tr$({\mathscr {F}}_{ij}{\mathscr {F}}_{ij})$ that is,

28 $$\begin{aligned} E_{\text{SO}}=\frac{\lambda }{4}\int dr{\mathscr {F}}_{ij}^a{\mathscr {F}}_{ij}^a, \end{aligned}$$

where $\lambda$ is a constant (on dimensional grounds $\lambda \sim p_F^{D-2}/m$, where $p_F$ is the Fermi momentum and $D>1$ is the dimension of space). Thus, the current due to the effective field yields,

29 $$\begin{aligned} j_i^a=-\frac{\delta E_{\text{SO}}}{\delta {\mathscr {A}}_i^a}. \end{aligned}$$

We may define the current induced by the screw dislocation as the Burgers current which can be expressed as, $j_{b^z}=-\frac{\delta E_{\text{SO}}}{\delta b^z}$. Further, the induced spin current, which we may define as the Burgers spin current can also be written as, $j_{S,b^z}=j_{b^z}(\uparrow )-j_{b^z}(\downarrow )$. Equation (27) is precisely the Yang-Mills magnetostatic equation. Intuitively, the results are analogous to the case of an external magnetic field. However, there is a difference in the spatial distribution of the currents induced by the screw dislocation.

**Figure 16:**
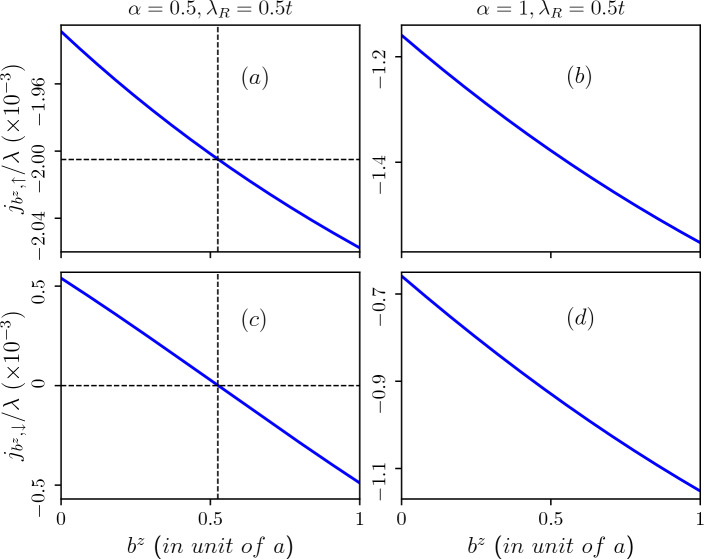
Burgers current as a function of the Burgers vector induced by the topological screw dislocation in presence of external magnetic field of $B_0=3$T. Upper panel: corresponds to $\uparrow$-spin (**a**) for $\alpha =0.5$ and (**b**) for $\alpha =1$. Lower panel: corresponds to $\downarrow$-spin (**c**) for $\alpha =0.5$ and (**d**) for $\alpha =1$. The Rashba spin-orbit coupling parameter is taken as $\lambda _R=0.5t$.

In Fig. 15, we depict the Burgers current for different spin sectors as a function of the Burgers vector corresponding to the lowest energy level of the valence band in the absence of an external magnetic field for $\alpha =0.5$ (refer to Fig. 15a ($\uparrow$-spin),c ($\downarrow$-spin), and for $\alpha =1$ (Fig. 15b ($\uparrow$-spin), d ($\downarrow$-spin)). These plots correspond to a fixed value of the Rashba spin-orbit coupling, namely, $\lambda _R=0.5t$. Equation (9) reveals that even in the absence of an external magnetic field, there exists an effective field due to the topological defect represented by the screw dislocation. This effective field contributes to the generation of Burgers current. For the scenario without an external magnetic field and $\alpha =0.5$ (refer to Fig. 15a,c), the Burgers current steadily decreases with an increase in the Burgers vector. Moreover, for $\alpha =1$ and only for the $\uparrow$-spin branch (Fig. 15b), the Burgers current undergoes a sign change at an approximate value of $b^z\sim 0.4$ (in unit of the lattice constant). This change in sign (can be interpreted as a back flow) of the current is attributed to the behaviour of the energy spectrum. Since the spectral properties undergo a change in slope and the current being proportional to that, a sign change occurs. One can clearly see that the variation in the $\uparrow$-spin Burgers current, $j_{b^z,\uparrow }$, and the $\downarrow$-spin Burgers current, $j_{b^z,\downarrow }$ with respect to $b^z$ are strikingly dissimilar and opposite in nature, rather than being equal and opposite, in the $\alpha =0.5$ scenario. These currents depict two circulating spin currents moving in opposite directions within the ring, potentially resulting in a chirality effect even without an external magnetic field. However, for $\alpha =1$, the situation significantly altered. Here, the chirality persists up to a certain threshold of screw dislocation ($b^z\sim 0.4$), beyond which both spin ($\uparrow$ and $\downarrow$) currents align in the same direction. Summarizing, the manifestation of the chiral current induced solely by the screw dislocation offers valuable insights with implications for future technological advancements.

Additionally, in Fig. 16, we depict the Burgers current as a function of the Burgers vector corresponding to the lowest energy level of the valence band in presence of an external magnetic field for $\alpha =0.5$ (refer to Fig. 16a ($\uparrow$-spin), c ($\downarrow$-spin) and $\alpha =1$ (refer to Fig. 16b ($\uparrow$-spin), d ($\downarrow$-spin). In the presence of an external magnetic field the bands corresponding to negative quantum number, and in particular $m=-6$ bands emerge as the lowest energy states, and these remain nearly constant with the Burgers vector. These plots are for a fixed value of the Rashba spin-orbit coupling ($\lambda _R=0.5t$). In the presence of a magnetic field, the sign-change feature of the Burgers current is observed for $\alpha =0.5$ only for the $\downarrow$-spin branch (refer to Fig. 16c), while there is a steady decrease in the Burgers current for $\alpha =1$ (refer to Fig. 16d). Here, chirality persists only for $\alpha =0.5$ up to a certain threshold of screw dislocation ($b^z\sim 0.5$), beyond which both spin currents align in the same direction. However, for $\alpha =1$ there is no chirality present in the system.

**Figure 17:**
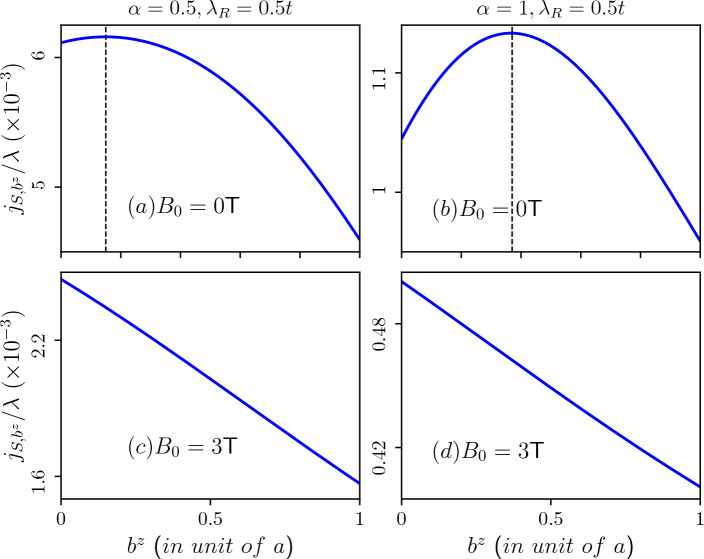
Burgers spin current as a function of the Burgers vector induced by the topological screw dislocation. Upper panel: in absence of external magnetic field (**a**) $\alpha =0.5$ and (**b**) $\alpha =1$. For $\alpha =0.5$ maximum Burgers spin current occurs at $b^z\sim 0.15$ (in unit of lattice constant) and for $\alpha =1$ maximum Burgers spin current occurs at $b^z\sim 0.4$ (in unit of lattice constant). Lower panel: in presence of external magnetic field of $B_0=3$T (**c**) $\alpha =0.5$ and (**d**) $\alpha =1$. The Rashba spin-orbit coupling parameter is taken as $\lambda _R=0.5t$.

Next,the Burgers spin current as a function of the Burgers vector for the scenarios mentioned above are illustrated in Fig. 17. The presence of the Rashba spin-orbit coupling in the system results in spin-split energy branches, leading to the emergence of Burgers spin current defined as the difference in Burgers current between the two spin branches. In the absence of an external magnetic field, we observe an initial increase in the Burgers spin current with the Burgers vector. Beyond a certain maximum value, it decreases for any value of $\alpha$. Notably, the Burgers vector corresponding to the maximum Burgers spin current increases with higher values of $\alpha$. For $\alpha =1$, the maximum of $j_{S,b^z}$ occurs at $b^z\sim 0.4$ (in unit of the lattice constant) (refer to Fig. 17b). Since the Burgers vector signifies the amount of screw dislocation in the system, maximizing the Burgers spin current would require large screw dislocations, and the behaviour is more for large values of $\alpha$. Moreover, the presence of an external magnetic field eliminates this upturn in $j_{S,b^z}$, as depicted in Fig. 17c,d, where the Burgers spin current steadily decreases with increasing $b^z$.

### Burgers dipole moment

**Figure 18:**
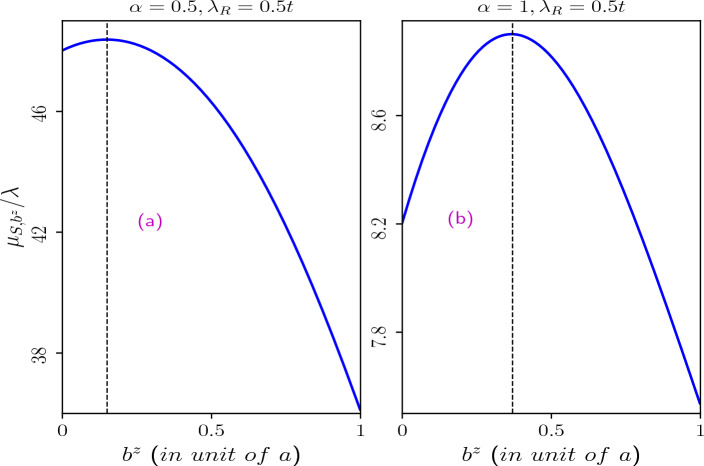
Burgers dipole moment as a function of the Burgers vector induced by the topological screw dislocation in absence of external magnetic field are shown. Here (**a**) for $\alpha =0.5$ and (**b**) for $\alpha =1$. The other parameters are taken as $\lambda _R=0.5t$ and $k\eta =0.3$.

Another interesting contribution to the dipole moment may come from the chiral current in the spin sector which arise even in the absence of an external magnetic field. The dipole moment, as expected, shows a non-monotonic behaviour (shown in Fig. 18) as a function of the strength of the dislocation (i.e. $b^z$) which hints towards an efficient ‘*operating region*’ for our system to yield large spin torque.

## Summary and conclusions

In summary, we have conducted a comprehensive investigation of the properties of Rashba spin-orbit coupling in the context of an $\alpha$-$T_3$ pseudospin-1 Fermionic Aharonov–Bohm quantum ring, considering the presence of a special type of topological defect. Our exploration covered aspects such as the energy spectrum, persistent currents, their dependencies on spin-orbit couplings, screw dislocations, and magnetic fields. Our key findings are as follows.

The introduction of the spin-orbit coupling parameter, $\lambda _R$, results in a spectrum composed of six bands, including two non-dispersive flat bands one for each spin, and four dispersive spin-split valence and conduction bands. The flat bands encompass a multitude of degenerate levels at zero-energy, which remain unaffected by applied magnetic fields. In the absence of a magnetic field, the energy levels in both the conduction and valence bands exhibit inverse dependence on the ring radius, denoted as *R*, and are independent of $\alpha$. Notably, $\uparrow$-spin energy levels are two-fold degenerate for $\alpha =1$, except for the $m=0$ level, while the $\downarrow$-spin bands are non-degenerate for all $\alpha$ values. The presence of a screw dislocation, serving as a topological defect, renders splitting of degeneracy, although other features remain unaltered. Under the influence of a perpendicular magnetic field, the energy levels deviate significantly from their usual *R*-dependence, showcasing behaviours of $\sim 1/R$ for small *R* and $\sim R$ for larger *R*. The presence of the topological defect introduces a topological term, effectively acting as a magnetic flux traversing the ring. This effective flux is the result of two contributions, one stemming from the topological nature of the defect and the other from the external magnetic flux.

The spin-split energy bands corresponding to different quantum number (*m*) exhibit distinct characteristics as a function of the Burgers vector in presence and absence of an external magnetic field. In the absence of an external magnetic field, the bands associated with quantum number $m=0$ represent the lowest energy states. Additionally, the energy levels demonstrate a nearly linear increase (or decrease) as a function of the Burgers vector. On the other hand, in the presence of an external magnetic field, the bands corresponding to negative quantum number, and in particular $m=-6$ bands emerge as the lowest energy states, and these remain nearly constant with the Burgers vector.

Furthermore, the persistent currents in both the spin and charge sectors exhibit periodic oscillations with a periodicity of $\Phi _0$, featuring distinct patterns corresponding to different $\alpha$ and $\lambda _R$ values. Notably, the presence of a topological defect shifts the phase of current oscillation by an amount equal to the strength of the defect. We have also derived equilibrium spin currents by combining the charge current contributions from different spin branches, underscoring the potential utility of our system in spintronic applications. Equilibrium spin currents are present for all $\alpha$ values, with a $\Phi =\Phi _0$ periodic behaviour. Similar to the charge persistent current, the presence of a topological defect shifts the phase of the oscillations in the spin current profile by an amount proportional to the defect.

The Burgers current exhibits an almost linear decrease with the Burgers vector, both in the presence and absence of an external magnetic field. However, without the external field, these current undergoes a sign change for $\alpha =1$, whereas in the presence of the external magnetic field, this sign change occurs for $\alpha =0.5$ at specific values of the Burgers vector. Additionally, the presence of distortion in the $\alpha$-$T_3$ lattice induces a chirality effect within the system, giving rise to an additional chiral current even in the absence of an external magnetic field. Moreover, in the absence of an external magnetic field, the Burgers spin current initially increases with an increase in the Burgers vector. After attaining a maximum, the current decreases with further increase in $b^z$ for all values of $\alpha$. However, this phenomenon disappears in the presence of an external magnetic field.

In conclusion, by adjusting the parameters $\alpha$, $k\eta$, $\Phi$, and $\lambda _R$, we have comprehensively shown the ability to manipulate the persistent currents, rendering them as controllable features in our system. To emphasize the role of the $\alpha$-$T_3$ ring system in spintronic devices, we have computed the induced dipole moment of the ring. Particularly, the contribution of the Burgers spin current yields useful information on the prospects of it being used for spintronic applications.

### Supplementary Information

Supplementary Information.

## Data Availability

The datasets used and/or analysed during the current study available from the corresponding author on reasonable request.
