# Supplementary material for: Screw dislocation in a Rashba spin-orbit coupled α-T3 Aharonov–Bohm quantum ring
Source: Sci Rep. 2024 May 16;14:11232. doi: 10.1038/s41598-024-61889-4 (PMC11099126; doi:10.1038/s41598-024-61889-4)
Supplement: Supplementary file 1 — Supplementary Information. [file 41598_2024_61889_MOESM1_ESM.pdf]

Supplementary Material for

Screw dislocation in a Rashba spin-orbit coupled α-T_3 Aharonov-Bohm quantum ring

Mijanur Islam[1*] and Saurabh Basu[1†]
[1] *Department of Physics, Indian Institute of Technology Guwahati, Guwahati-781039, Assam, India.*

The scattering of an electron by a screw dislocation is analogous to the scattering by an Aharanov-Bohm flux, β in the quantum ring. The same is applicable to any gauge field, such as the one that we have in our system, namely, the field arising due to the screw dislocation. Let us elaborate it as follows. Consider an incident plane wave with a wave vector k propagating along the z-direction and is parallel to the external magnetic flux. The wave undergoes a phase shift due to the presence of the Aharonov-Bohm flux. In polar coordinates, the scattering phenomenon in two dimensions is described by the Schrödinger equation[1–3],

$$\left[\frac{\partial^2}{\partial r^2} + \frac{1}{r}\frac{\partial}{\partial r} + \frac{1}{r^2}\Big(\frac{\partial}{\partial \theta} + i\beta\Big)^2 + k^2\right]\psi(r) = 0. \tag{1}$$

The general solution of Eq. (1) is given by,

$$\psi(r) = \sum_{m=-\infty}^{\infty} a_m e^{im\theta} J_{|m+\beta|}(kr) \tag{2}$$

where $J_{|m+\beta|}(kr)$ is the Bessel function. Prior to scattering, we have to match the incoming wave (before scattering) with the incoming asymptotic polar component of the solution. Let the incoming wave is,

$$\psi_{inc} \sim \sqrt{\frac{1}{2\pi kr}} \sum_{m=-\infty}^{\infty} (-i)^m e^{im\theta} e^{-ikr} e^{im\pi/2} e^{i\pi/4}. \tag{3}$$

The polar component of the incoming wave which is useful for our purpose, is given by,

$$\big(\psi_{inc}\big)_\theta \sim \sqrt{\frac{1}{2\pi kr}} \sum_{m=-\infty}^{\infty} a_m e^{im\theta} e^{-ikr} e^{i|m+\beta|\pi/2} e^{i\pi/4} e^{i\beta\theta_k} \tag{4}$$

where θ_k is the angle of propagation. The matching condition requires that $a_m = e^{-i|m+\beta|\pi/2}e^{-i\beta\theta_k}$. The same is true for the outgoing wave as well which is expressed as,

$$\psi_{out} \sim \sqrt{\frac{1}{2\pi kr}} \sum_{m=-\infty}^{\infty} (-i)^m e^{im\theta} e^{ikr} e^{-im\pi/2} e^{-i\pi/4} + f(\phi)\frac{e^{ikr}}{\sqrt{r}}. \tag{5}$$

Again, the polar component of the outgoing wave is,

$$\big(\psi_{out}\big)_\theta \sim \sqrt{\frac{1}{2\pi kr}} \sum_{m=-\infty}^{\infty} e^{im\theta} e^{ikr} e^{-i|m+\beta|\pi/2} e^{-i\pi/4} e^{-i\beta\theta_k}. \tag{6}$$

The outgoing wave is the superposition of the incoming (unscattered) wave and of the one with a scattering amplitude $f(\phi)$. The phase shift, δ_m, in the scattering is obtained by comparing the two outgoing plane waves of Equations (5) and (6) and has a form,

$$\delta_m = -\frac{\pi}{2}\Big(|m+\beta| - |m|\Big) \tag{7}$$

β in Eq. (7) contains the scattering effects. For the screw dislocation, we have used $\beta = k\eta$ in our calculations.

---

* Electronic address: mislam@iitg.ac.in

[†] Electronic address: saurabh@iitg.ac.in

[1] C. R. Hagen, *Aharonov-Bohm scattering of particles with spin*, Phys. Rev. Lett. **64**, 503 (1990).
[2] C. R. Hagen, *Aharonov-Bohm scattering amplitude*, Phys. Rev. D **41**, 2015 (1990).
[3] V. Parente, G. Campagnano, D. Giuliano, A. Tagliacozzo, and F. Guinea, *Topological Defects in Topological Insulators and Bound States at Topological Superconductor Vortices*, Materials, **7**, 1652 (2014).
